# The benefit of choice on task performance: Reduced difficulty effects in free-choice versus forced-choice tasks

**DOI:** 10.3758/s13421-024-01641-5

**Published:** 2024-10-07

**Authors:** Victor Mittelstädt, Ian Grant Mackenzie, Denise Baier, Lili Goetz, Pia Wittbecker, Hartmut Leuthold

**Affiliations:** Department of Psychology, University of Tübingen, Schleichstraße 4, 72076 Tübingen, Germany

**Keywords:** Voluntary task switching, Cognitive control, Flexibility, Multitasking

## Abstract

We investigated how self-determined (free) versus imposed (forced) choices influence task performance. To this end, we examined how changes in perceptual and central decision-processing difficulties affect task performance in an environment where free-choice and forced-choice tasks were intermixed. In Experiments 1 (*N* = 43) and 2 (*N* = 42), perceptual processing difficulty was varied by altering colored dot proportions (easy vs. hard color discrimination task). In Experiment 3 (*N* = 58), decision-processing difficulty was adjusted by changing the rotation degree of letters (easy vs. hard letter rotation task). Across all experiments, both free-choice and forced-choice performance were more impaired with harder stimuli, but this effect was generally less pronounced in freely chosen tasks. Specifically, this was evident from significant interactions between processing mode (free vs. forced) and difficulty (easy vs. hard) in the mean reaction times (RTs) for the tasks with the difficulty manipulation. Thus, processing in free-choice tasks is generally less affected by environmental changes (i.e., variation in information difficulties). We discuss how the benefit of self-determined choices over imposed choices can be explained by motivational and performance-optimization accounts, while also considering the finding that participants adjusted their task choices toward tasks with easier stimuli (i.e., significant main effect of task difficulty on choosing the task with the difficulty manipulation). Specifically, we discuss how having control over task choices might lead to more stable information processing and allow people to choose more difficult tasks when this increased difficulty has a relatively small impact on their performance.

## Introduction

We are constantly surrounded with multiple sources of information associated with different task goals. One major goal in cognitive psychology is to unveil the underlying mental mechanisms that enable one to process information in an adaptive, goal-directed manner. Given that individuals often freely decide which information to attend to and which tasks to select, it is crucial to address the question of how personal control over tasks choices influences our behavior (e.g., Deci et al., 2008; Leotti et al., 2010; Chambon et al., 2020; Mittelstädt et al., 2024). Specifically, this raises the question of whether information is processed in a similar manner or, at least partially, in a distinct manner according to self-determined (free-choice) and imposed (forced-choice) task goals. The present study aims to address this question by investigating how the difficulty of processing task-specific information influences task processing in an environment where free-choice and forced-choice tasks are intermixed.

Free task choice behavior can be studied through various paradigms (e.g., Mittelstädt et al., 2019; Arrington and Logan, 2005; Braem, 2017; Lien and Ruthruff, 2008; Brosowsky and Egner, 2021; Vandierendonck et al., 2012; Dreisbach and Jurczyk, 2022; Brüning et al., 2021; Imburgio and Orr, 2021; Fintor et al., 2020). In the present study, we employ the hybrid free-forced paradigm, wherein participants encounter two stimuli (e.g., a letter and a colored square) associated with two independent task sets in free-choice trials, but only one task stimulus (e.g., only a letter) in forced-choice trials (e.g., Mendl and Dreisbach, 2022; Jurczyk et al. , Qiao et al., 2023; Mittelstädt et al., 2023; Mittelstädt et al., 2022). Within this paradigm (as well as others), researchers primarily focus on the outcomes of experimental manipulations on *task choice* in free-choice trials, with a general agreement that both cognitive and environmental factors can influence task choices (e.g., Arrington, 2008; Mittelstädt et al., 2019; Dreisbach and Fröber, 2019; Mittelstädt et al., 2024). For example, participants tend to favor repeating the previously performed task when given a choice, suggesting that their choices are sensitive to the cognitive limitations encountered during task-switching (e.g., switch-related reconfiguratory processes, cf. Rogers and Monsell, 1995; Kiesel et al., 2010; Koch et al., 2018; Meiran, 2008; Jersild, 1927; Vandierendonck et al., 2010; Kang and Chiu , Kang and Chiu ) Furthermore, participants tend to choose the task associated with the first of two sequentially presented stimuli, suggesting that their choices are sensitive to environmental changes (e.g., Arrington, 2008; Mittelstädt et al., 2024). Together, these findings are thought to reflect adaptive behavior, where cognitive and environmental constraints jointly guide task choice to improve performance in free-choice trials (e.g., Mittelstädt et al., 2018; Mittelstädt et al., 2024; Mittelstädt et al., 2024). However, it remains unclear whether and how task processing in a free-choice mode differs from that in a forced-choice mode, as researchers typically do not directly compare *task performance* between these two modes.

**Fig. 1:**
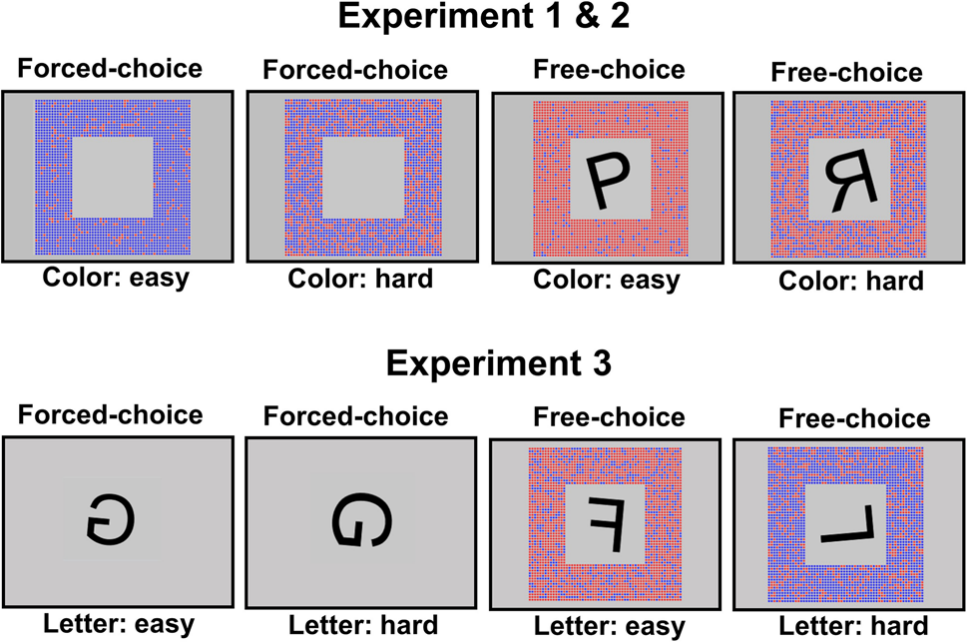
Possible stimulus displays in Experiments 2 and 3. The stimulus displays in Experiment 1 were similar, except that only normal (non-mirrored) letters were presented. The task in Experiment 1 was to classify whether the letter was a vowel or consonant and in Experiments 2 and 3, the task was to determine whether the letter was mirrored or not

In the present study, we aim to shed light on this issue by investigating how changes in processing difficulty affect free- and forced-choice performance. Specifically, in Experiments 1 and 2, the perceptual difficulty of stimuli for one of the two tasks (i.e., easy vs. hard color task stimulus) was varied in a random manner within blocks. In the free-choice trials, participants could voluntarily decide whether they wanted to perform the color task (i.e., classifying whether there were more red or blue dots, cf. Fig. 1) or the letter task (e.g., determining whether the letter is mirrored or normal) and in forced-choice trials, only one of the two stimuli (color or letter) was presented. In Experiment 3, we tested the effect of a different difficulty manipulation related to a letter rotation task on free-choice and forced-choice performance. For this task, participants had to decide whether a rotated letter was mirrored or normal, with the rotation being either a small or large angle, making processing relatively easy or hard. There is robust evidence indicating that mental rotation occurs during a central stage of processing and, if at all, scarcely overlaps with preceding perceptual processing (e.g., Ruthruff et al., 1995; Ruthruff and Miller, 1995). Thus, using this decision difficulty manipulation in Experiment 3 in addition to the perceptual difficulty manipulation associated with the color task in Experiments 1 and 2 allows us to study the effects of two fundamentally different difficulty manipulations on task processing. In line with previous findings, we expected to replicate the pattern of poorer task performance (in terms of reaction times and/or error rates) under a forced-choice mode when the task stimulus was difficult compared to when it was easy to process (e.g., Mittelstädt et al., 2022; Ruthruff and Miller, 1995). However, it is unclear to what extent the effect of a difficulty manipulation on processing is similarly observed when participants freely decide on a task.

On the one hand, there are both theoretical and empirical reasons to hypothesize that in the present study, the impact of task difficulty on processing (e.g., perception or central processing) might be smaller in a free-choice mode than in a forced-choice mode, resulting in an interaction between processing mode and task difficulty in task performance. Specifically, consider that self-determination theory emphasizes the importance of autonomy (e.g., Deci et al., 1989; Ryan and Deci, 2000; Deci et al., 2008). Relatedly, Bandura’s concept of agency emphasizes the importance of self-efficacy and the sense of being in control of one’s actions (e.g., Bandura, 2001). When people are allowed to choose freely, they exercise their agency, which can enhance motivation, engagement, and commitment, resulting in greater effort and more stable processing. For example, Bouzidi et al. (2022) showed that choosing the colors of letters in a memory task, rather than having a color assigned, can increase goal-directed commitment and thus justify higher effort and improve performance (i.e., the number of correctly remembered letters) when task difficulty is unclear (i.e., no information about the upcoming workload and task duration is available). Thus, in the present study, freely choosing a task with unpredictably varying difficulty, rather than being forced to perform it, might also lead to greater effort intensity. This, in turn, could reduce the impact of task difficulty in free-choice compared to forced-choice processing. Furthermore, self-determination theory emphasizes the importance of competence – the drive to experience mastery and effectiveness (e.g., Deci et al., 1989; Ryan and Deci, 2000). When people are allowed to choose freely, they can explore different strategies and approaches that align better with their strengths. This increased flexibility in a free-choice mode, compared to an externally controlled forced-choice mode, can be particularly beneficial when tackling more difficult tasks. Moreover, better alignment of self-chosen tasks with one’s current cognitive state can enhance feelings of competence, which in turn can further improve performance on more difficult tasks. Indeed, having the possibility to choose can enhance memory (e.g., Katzman and Hartley, 2020; Murty et al., 2015), improve learning (e.g., Cordova and Lepper, 1996; Reber et al., 2018), and increase the time spent on activities like puzzle solving (e.g., Zuckerman et al., 1978).

On the other hand, one might question whether such benefits of choice are observed when choosing between relatively simple cognitive tasks, as in the present study, suggesting that there might instead be no differences in the effects of difficulty on performance as a function of processing mode. Such a potential pattern (i.e., a main effect of difficulty but no interaction) also seems supported by a similar influence on a range of cognitive effects in different free-choice and forced-choice paradigms. (e.g., Flowerday and Schraw, 2003; Janczyk et al., 2015; Pfeuffer et al., 2023; Hackländer et al., 2023; Bermeitinger and Hackländer, 2018). Moreover, while several recent studies have shown that personal choice versus external task assignment can affect effort-related physiological measures (such as the cardiac pre-ejection period) (e.g., Bouzidi et al., 2022; Framorando et al., 2024; Framorando et al., 2023; Gendolla et al., 2021; Falk et al., 2022), only a few of these studies have also demonstrated that this can lead to changes in task performance (e.g., Bouzidi et al., 2022; Gendolla et al., 2021). In addition to the somewhat unclear empirical effects of personal control on task performance, it is theoretically useful to consider that free-choice trials involve the additional process of selecting a task, whereas forced-choice trials do not. Choosing a task is in itself demanding, and may use the same resources required for task processing (e.g., Kiesel and Dignath, 2017; Vohs et al., 2018; Schwartz, 2000; Naefgen et al., 2018; Janczyk et al., 2015). Consequently, participants under a free-choice mode might be particularly challenged to allocate additional resources to process a hard stimulus as some of the limited resources have just been, or are still being, used to choose a task. Thus, there are several reasons that question whether there will indeed be a benefit of free-choice on task performance in terms of reduced difficulty effects.

To better understand how the difficulty manipulation affects behavior, we also analyzed its impact on task choices during free-choice trials. In a previous study involving a perceptual difficulty manipulation, participants exhibited a difficulty choice bias, preferring to select the color task with easier stimuli over harder ones (Mittelstädt et al., 2022). Such a choice bias might explain – or at least contribute to – potential differences in task performance between free-choice and forced-choice trials. For example, as difficulty varies unpredictably across trials, participants need to engage in some processing to adjust their behavior during a trial. Thus, it appears that participants can at least initially process the stimuli for each task in parallel and then flexibly adapt their choice behavior based on which task has made the most progress. Consequently, this adaptive task choice behavior may lead participants to select the difficult task only when it impairs performance to a lesser degree, resulting in reduced difficulty effects in free-choice compared to forced-choice modes. Furthermore, as we will discuss in more detail in the General discussion, investigating how participants adapt their choice behavior to a difficulty manipulation affecting different processing stages (perceptual stage via discriminability and decision stage via mental rotation) can provide deeper insights into the locus of voluntary choices within the information processing stream.

## Experiment 1

We first provide a reanalysis of the data of Experiment 4 of Mittelstädt et al. (2022). As mentioned earlier, these authors reported the effects of the perceptual task difficulty manipulation on task choice behavior and forced-choice processing, but the effects on free-choice processing (and its comparison to forced-choice processing) were not investigated. The basic tasks used in this experiment were a letter categorization task and a color categorization task (cf. Fig. 1). In the letter task, participants had to decide whether a letter was a vowel or a consonant. In the color task, participants had to determine whether there were more red or blue dots. Crucially, the difficulty within the color task was randomly manipulated across trials by varying the ratio of differently colored dots (i.e., color ratio easy: 90% vs. 10%; color ratio hard: 62.5% vs. 37.5%). Furthermore, it was randomly varied across trials whether participants could freely choose between the letter and color tasks or were required to perform a specific task. Specifically, in free-choice trials, two stimuli were presented, and participants were informed that in these trials they could choose which task to perform. In forced-choice trials, only one stimulus was displayed, and participants were instructed to perform the task associated with the presented stimulus. We first present the results of how the randomly varying perceptual difficulty of color stimuli influences participants’ task choices in free-choice trials (cf. Mittelstädt et al., 2022). Next, we compare the effects of the difficulty manipulation on task performance in free-choice versus forced-choice trials when responding to the task with varying difficulty (i.e., the color task).

### Method

#### Sample size justification

As Experiment 1 was a reanalysis of a previously published study, the sample size of 60 participants was already determined. The effects obtained in this experiment were quite large ($\eta ^2_p$ = 0.36 for the effect of color difficulty on the percentage of color task choices and $\eta ^2_p$ = 0.37 for the interaction of processing mode and color difficulty on mean RTs). According to a power analysis using the R-package Superpower (cf. Lakens & Caldwell, 2021), a sample size of at least 18 participants would have already provided 80% power with a significance level of 5% to detect similar effect sizes. However, we decided and preregistered a larger, somewhat arbitrary sample size of *N* = 50 for Experiments 2 and 3 to account for potential dropouts and the possibility that the effects might be smaller with another difficulty manipulation. As described in the section on Experiment 3, we also decided during the review process to double the sample size in this experiment. We further conducted sensitivity analyses with the final sample sizes related to the interaction between processing mode and task difficulty separately for each experiment. These analyses revealed that we had 80% power at a significance level of 5% to detect effects of at least $\eta ^2_p = 0.17$ in Experiment 1 (*N* = 43), $\eta ^2_p = 0.17$ in Experiment 2 (*N* = 42), and $\eta ^2_p = 0.13$ in Experiment 3 (*N* = 58).

#### Participants

Sixty participants were tested online, but the data from 17 participants had to be excluded (cf. data preparation section). All participants were recruited via Prolific and received monetary compensation. Consequently, the final sample comprised 43 people (33 women, 37 right-handed), with ages ranging from 18 to 57 years (M = 27.67).

#### Apparatus and stimuli

This and the following experiments were conducted online using the JavaScript library jsPsych (De Leeuw, 2015). All visual stimuli were presented on a grey background. A centrally positioned plus sign served as fixation point. For the letter task, participants had to decide whether the letter was a vowel or consonant by pressing a left vs. right key. A specific letter was randomly selected from the set of letters “A”, “E”, “G”, “I”, “K”, “M”, “R”, “U”. For the color task, participants had to classify whether the dots surrounding the letter were primarily red or blue by pressing a left vs. right key. Critically, the classification was either easy or difficult because of the proportions of the two colored dots (i.e., color ratio easy: 90% vs. 10%, color ratio hard: 62.5% vs. 37.5%). Responses for each task were made with the index and middle finger of the same hand on the ‘Q”,“W”,“O”, and“P” keys. The task-to-hand mapping was counterbalanced across participants whereas the specific response-finger mappings were randomly selected for each participant.

#### Procedure

Participants were tested in eight blocks of 104 trials per block. Each block consisted of 50% free choice (i.e., 52 trials with color and letter task stimuli) and 50% forced choice (i.e., 26 trials with only color and 26 trials with only letter stimuli) randomly intermixed trials. Furthermore, when a color stimulus was presented, it was easy in half of the trials and hard in the other half.

Participants were instructed that in free-choice trials (i.e., when both stimuli were presented), they were free to perform whichever task they wanted. However, in forced-choice trials (i.e., when only one stimulus appeared) they had to perform the task related to the stimulus (see Fig. 1).

At the beginning of each trial, the fixation cross appeared on the screen for 500 ms. In free-choice trials, both color and letter stimuli were displayed immediately at the offset of the fixation cross. In forced-choice trials, only one stimulus (i.e., letter or color) was presented. The stimulus (stimuli) remained on the screen until the participant responded. Following correct responses, a blank screen was presented for 750 ms before the next trial started. In case of an error, an additional error screen was presented for 3 s (first practice block: 4 s) indicating that an error had been made and repeating the S-R mappings for the two tasks.

#### Data preparation

We followed the same data preparation procedure that was preregistered for Experiments 2 and 3 to make the results more comparable within this manuscript. The first block and the first trial of each block were excluded from all analyses. Furthermore, we excluded trials with reaction times (RTs) less than 200 ms (< 0.1%) or more than 3000 ms (1.4%) and post-error (4.1%) trials from any analyses.^1^ For task choice (and RT) analyses, we additionally excluded any erroneous trials (4.1%).

After our data trimming procedure, we examined whether participants followed any consistent global task choice strategies in free-choice trials (i.e., always selecting the same task or always selecting the same or different task that was performed in the previous trial). Following our preregistration, we excluded the data of eight participants who selected one of the two tasks in >95% of trials and/or switched or repeated tasks in >95%.^2^

### Results and discussion

#### Task choice

**Fig. 2:**
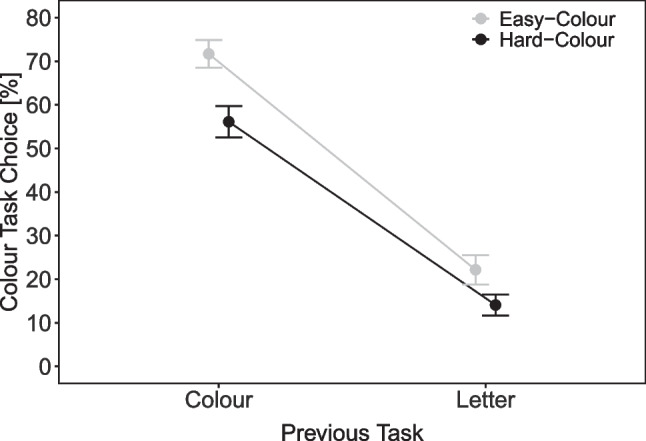
Task choice in Experiment 1. Note. Percentage of color task responses as a function of previous task (color, letter) and color task difficulty (easy, hard) in Experiment 1

Overall, there were more color-task choices when the color stimulus was easy (44.8%) than hard (33.3%), *t*(42) = 4.94, $p < .001, d$ = 0.75. An ANOVA with the factors previous task (color, letter) and color task difficulty (easy, hard) on the mean percentages of color task choices revealed significant main effects of color difficulty, *F*(1, 42) = 23.91, $p < .001, \eta ^2_p$ = .36, and previous task (reflecting a repetition bias), *F*(1, 42) = 269.88, $p < .001, \eta ^2_p$ = .87. Moreover, the interaction was significant, *F*(1, 42) = 12.77, $p < .001, \eta ^2_p$ = .23. As can be seen in Fig. 2, this interaction indicated that there were notably more color task choices for easy color stimuli than for hard ones when the preceding task was the color task (71.7% vs. 56.11%) than the letter task (22.2% vs. 14.1%, with *p*s < .001 for both pairwise comparisons).

#### Task performance – Effects of color difficulty under free and forced color-task processing

**Fig. 3:**
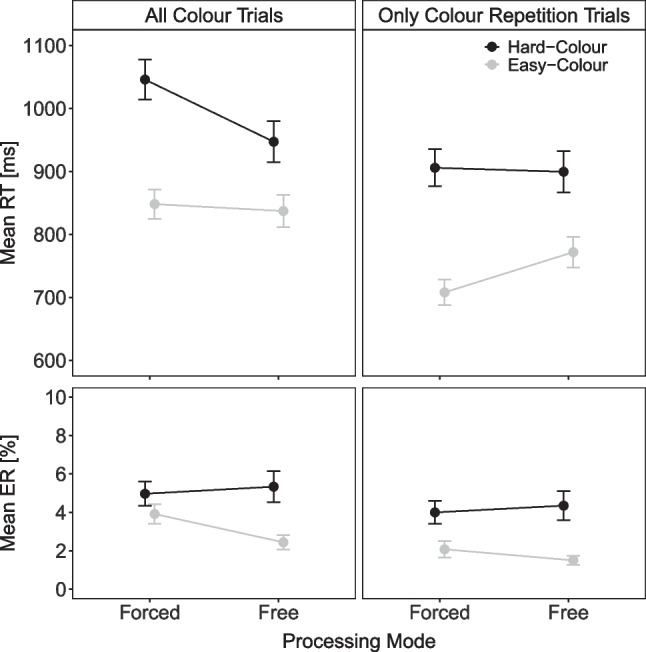
Task performance in Experiment 1. Note. Mean reaction time (RT) and mean error rate (ER) of color task responses as a function of color task difficulty (easy, hard) and processing mode (free, forced), including all color task trials (*left column*) and only color task trials preceded by color task trials (*right column*)

Figure 3 shows the mean RT and mean percentage error (PE) for color task responses as a function of color task difficulty (easy, hard) and processing mode (free, forced). A 2$\times$2 within-subject ANOVA on mean RT revealed that all effects were significant: The main effect of color difficulty indicated faster responses with easy than hard color stimuli (843 vs. 996 ms), *F*(1, 42) = 116.75, $p < .001, \eta ^2_p$ = .74. The main effect of processing mode reflected faster free than forced color task responses (892 vs. 947 ms), *F*(1, 42) = 11.65, $p = .001, \eta ^2_p$ = .22. The interaction indicated that the color difficulty effect was larger under a forced- than free-choice processing mode (198 vs. 99 ms), *F*(1, 42) = 24.20, $p < .001, \eta ^2_p$ = .37. The ANOVA on mean PE also revealed a significant main effect of color with less erroneous responses with easy than hard color stimuli (3.2% vs. 5.2%), *F*(1, 42) = 20.51, $p < .001, \eta ^2_p$ = .33. Moreover, a significant interaction indicated a smaller difficulty effect under forced- than free-choice processing, *F*(1, 42) = 4.64, *p* = .037, $\eta ^2_p$ = .10.^3^

The task choice results indicated that participants had a preference for task repetitions. Consequently, in the analyses of color task performance, free-choice trials featured a greater number of repetitions compared to switch trials in comparison to forced-choice trials. Hence, the larger color task difficulty RT effect observed under forced-choice processing might be attributed to the cognitive resources required to initiate a task switch. To explore this possibility, we reran the performance analyses, focusing exclusively on color task repetition trials (see Fig. 3). Please note that we had not thought about this analysis before reanalyzing the data from Experiment 1 or conducting the preregistered Experiments 2 and 3. Therefore, these findings should be considered exploratory. For mean RT, the ANOVA revealed again a significant main effect of color difficulty with faster responses with easy than hard color stimuli (740 vs. 903 ms), *F*(1, 42) = 123.54, $p < .001, \eta ^2_p$ = .75. The main effect of processing mode indicated slower responses under a free- than forced-choice processing mode (836 vs. 806 ms), *F*(1, 42) = 4.85, *p* = .003, $\eta ^2_p$ = .10. The interaction was significant reflecting again a reduced color difficulty effect under a free- than forced-choice processing mode (127 vs. 198 ms). *F*(1, 42) = 9.74, *p* = .003, $\eta ^2_p$ = .19. For mean PE, there was only a significant main effect of color task difficulty with fewer errors with easy than hard color stimuli (1.8% vs. 4.2%), *F*(1, 42) = 21.60, $p < .001, \eta ^2_p$ = .34 (all other $p > .286, \eta ^2_p<$ .04). Thus, these additional results suggest that the more pronounced color difficulty effect under a forced-choice processing mode, compared to a free-choice mode, persists even when considering only repetition trials (and when there is no evidence of a speed–accuracy tradeoff as in the overall analysis).

## Experiment 2

The reanalysis in Experiment 1 of the data obtained by Mittelstädt et al. (2022) indicate that the color difficulty RT effect was reduced in free-choice processing compared to forced-choice processing. The goal of Experiment 2 was to determine whether this pattern could be replicated in a preregistered experiment. Therefore, we employed the same perceptual difficulty manipulation. However, in preparation for the subsequent Experiment 3, we replaced the letter vowel-consonant task with a letter rotation task. In this task, participants had to determine whether the orientation of a rotated letter was normal or mirrored. Note that the rotation – and thus the difficulty of the letter task – remained consistent across trials in this experiment.

### Method

#### Participants

Fifty participants were tested online, but the data of eight participants had to be excluded (cf. preregistration and data preparation section). Participants were primarily recruited from the pool of psychology students at the University of Tübingen via internal departmental e-mail lists or social media and could receive course credits as compensation. Consequently, the final sample comprised 42 people (33 women, 37 right-handed), with ages ranging from 18 to 57 years (M = 24.02).

#### Apparatus, stimuli, and procedure

In this experiment, the only change was that, for the letter task, participants had to determine whether the letter was mirrored or normal by pressing either the left or right key: A specific letter was randomly selected from the set of letters “F”, “G”, “L”, “P”, “R” in each trial and this chosen letter was randomly tilted to the left or right at an angle of 5$^{\circ }$. Thus, each participant was presented with five different letters throughout the experiment, which were either mirrored or not.

#### Data preparation

As preregistered, the first block and the first trial of each block were again excluded from all analyses. Furthermore, we excluded trials with RTs less than 200 ms (<0.1%) or more than 3000 ms (0.8%) and post-error (4.8%) trials from any analyses. For task choice (and RT) analyses, we additionally excluded any erroneous trials (4.8%). Following our preregistration, we additionally excluded the data of eight participants who selected one of the two tasks in >95% of trials and/or switched or repeated tasks in >95%.

### Results and discussion

#### Task choice

**Fig. 4:**
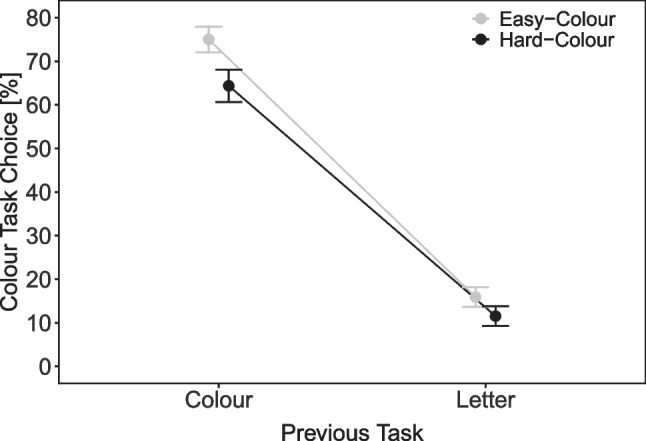
Task choice in Experiment 2. Note. Percentage of color task responses as a function of previous task (color, letter) and color task difficulty (easy, hard) in Experiment 2

Overall, there were more color-task choices when the color stimulus was easy (42.6%) than hard (35.6%), *t*(41) = 6.05, $p < .001, d$ = 0.93. Figure 4 shows the percentage of color task choices as a function of previous task (color, letter) and color task difficulty (easy, hard). In addition to a significant main effect of color difficulty, *F*(1, 41) = 37.92, $p<$ .001, $\eta ^2_p$ = .48, there was a significant main effect of previous task indicating a strong repetition bias, *F*(1, 41) = 319.02, $p<$ .001, $\eta ^2_p$ = .89. Moreover, the interaction was significant, *F*(1, 41) = 11.26, *p* = .002, $\eta ^2_p$ = .22. As can be seen in Fig. 4, the effect of difficulty in biasing color task choices was more pronounced when the preceding task was the color task rather than the letter task (with *p*s < .001 for both pairwise comparisons).

**Fig. 5:**
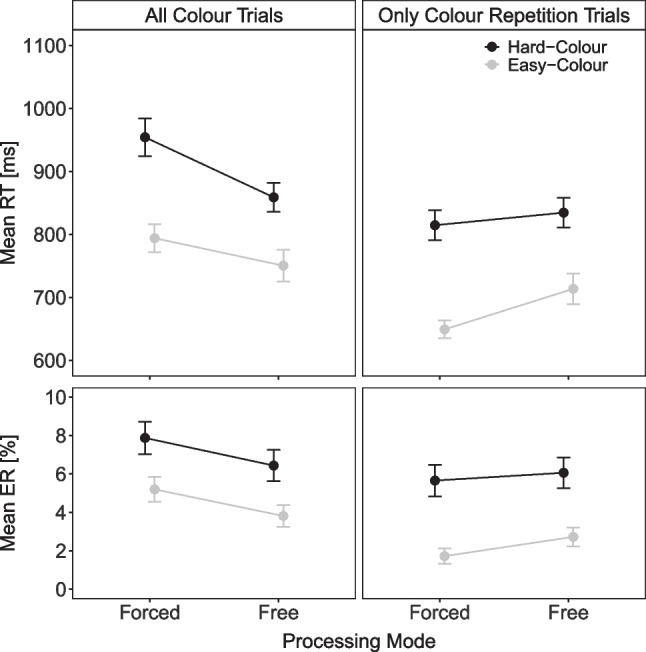
Task performance in Experiment 2. Note. Mean reaction time (RT) and mean percentage error (PE) of color task responses in Experiment 2 as a function of color task difficulty (easy, hard) and processing mode (free, forced), including all color task trials (left column) and only color task trials preceded by color task trials (right column)

#### Task performance – Effects of color difficulty under free and forced color-task processing

Figure 5 shows the mean RT and mean PE for color task responses as a function of color task difficulty (easy, hard) and processing mode (free, forced). A 2$\times$2 within-subject ANOVA on mean RT revealed that all effects were significant: The main effect of color difficulty indicated faster responses with easy than hard color stimuli (772 vs. 907 ms), *F*(1, 41) = 185.98, $p < .001, \eta ^2_p$ = .82. The main effect of processing mode reflected faster free than forced color task responses (805 vs. 874 ms) *F*(1, 41) = 10.53, *p* = .002, $\eta ^2_p$ = .20. The interaction indicated that the color difficulty effect was smaller under a free- than forced-choice processing mode (109 vs. 160 ms), *F*(1, 41) = 10.60, *p* = .002, $\eta ^2_p$ = .21. The ANOVA on mean PE also revealed a significant main effect of color with less erroneous responses with easy than hard color stimuli (4.5% vs. 7.2%), *F*(1, 41) = 18.21, $p < .001, \eta ^2_p$ = .31. Moreover, the significant main effect of processing mode reflected less errors for free than forced color task responses (5.1% vs. 6.5%), *F*(1, 41) = 4.88, *p* = .033, $\eta ^2_p$ = .11. The interaction was not significant (*p* = .963, $\eta ^2_p<$ .01).

The results were quite similar when we reran the performance analyses using only color task repetition trials (see Fig. 5). For mean RT, the ANOVA revealed again a significant main effect of color difficulty with faster responses with easy than hard color stimuli (681 vs. 824 ms), *F*(1, 41) = 166.86, $p < .001, \eta ^2_p$ = .80. The main effect of processing mode was also significant, but the effect was in the opposite direction with slower responses under a free- than forced-choice processing mode (774 vs. 732 ms), *F*(1, 41) = 7.77, *p* = .008, $\eta ^2_p$ = .16. The interaction was significant reflecting again a reduced color difficulty effect under a free- than forced-choice processing mode (121 vs. 166 ms), *F*(1, 41) = 4.24, *p* = .046, $\eta ^2_p$ = .09. For mean PE, there was only a significant main effect of color task difficulty with less errors with easy than hard color stimuli (2.2% vs. 5.9%), *F*(1, 41) = 34.64, $p < .001, \eta ^2_p$ = .46 (all other $p > .149, \eta ^2_p<$ .06).

## Experiment 3

In Experiment 2, the color difficulty effect on color task performance was again more pronounced under a forced-choice processing mode than under a free-choice mode. The goal of Experiment 3 was to determine which of these findings would generalize using a difficulty manipulation that affects processing at a later stage (i.e., response selection), namely, mental rotation. Thus, in this experiment, the difficulty of the letter task was adjusted by altering the degree of rotation of the letter stimulus, while the difficulty of the color task remained constant.

### Method

We tested 100 people online, but we excluded the data of 40 participants who selected one of the two tasks in >95% of trials and/or switched or repeated tasks in >95%. It should be noted that the primary reason for exclusion (in 32 cases) was a strong preference for the color task. Moreover, two additional participants were excluded due to accuracy below 80%. The final 58 participants (52 right-handed, 33 female) ranged in age from 18 to 57 years (M = 26.1). Participants were recruited via Prolific or from the pool of psychology students at the University of Tübingen and could receive either course credits or monetary compensation.

Note that we initially planned (and preregistered) to test only 50 participants. However, we decided to double the sample size during the review process due to the high exclusion rate (24 out of 50 participants were excluded from the initial sample). The reported results for the final sample were very similar to those for the initial sample, except that the effect of letter difficulty on task choices (*t*(25) = 1.25, *p* = .223, *d* = 0.24) was not significant in the initial sample. As one might argue that we doubled the original sample size after the initial results were known and then applied the same analyses, the *p* value of the final analyses is biased and needs to be corrected for double testing. However, even when correcting the critical significance level to $\alpha = 0.050/2 = 0.025$, the interpretation of the results does not change. However, the descriptive patterns of these and all other effects were similar, suggesting that the lack of significance in the initial sample was due to insufficient power.

The final 58 participants (52 right-handed, 33 female) ranged in age from 18 to 57 years (M = 26.1). Participants were recruited via Prolific or from the pool of psychology students at the University of Tübingen and could receive either course credits or monetary compensation.

#### Apparatus, stimuli, and procedure

The apparatus, stimuli, and procedure were the same as in Experiment 2, with the only difference being that the color task stimuli had a fixed difficulty (color task ratio of 72.5% to 27.5%) while the difficulty of the letter task varied randomly between trials by altering the rotation angle (letter easy: 5$^{\circ }$; letter hard: 95$^{\circ }$). Note that the fixed ratio level of the color task in this experiment was roughly between the easy and hard ratio levels used in Experiments 1 and 2.

#### Data preparation

We followed the same data preparation procedure as in Experiments 1 and 2. Thus, trials with RTs less than 200 (<0.1%) ms or more than 3000 ms (1.0%) and post-error trials (4.9%) were excluded from all analyses. For task choice (and RT) analyses, we additionally excluded any erroneous trials (4.9%).

### Results and discussion

#### Task choice

**Fig. 6:**
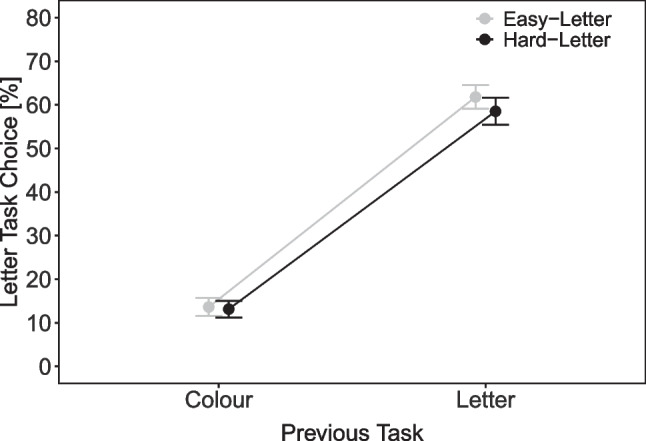
Task choice in Experiment 3. Note. Percentage of letter task responses as a function of previous task (color, letter) and letter task difficulty (easy, hard) in Experiment 3

Overall, there were more letter-task choices when the letter stimulus was easy (33.8%) than hard (31.9%), *t*(57) = 2.50, *p* = .015, *d* = 0.33. Figure 6 shows the percentage of letter task choices as a function of previous task (color, letter) and letter task difficulty (easy, hard). There was a significant main effect of previous task, *F*(1, 57) = 256.27, $p < .001, \eta ^2_p$ = .82, indicating a strong repetition bias. The main effect of letter difficulty was also significant *F*(1, 57) = 5.30, *p* = .025, $\eta ^2_p$ = .09. The interaction was not significant, *F*(1, 57) = 2.80, *p* = .100, $\eta ^2_p$ = .05. As can be seen in Fig. 6, on a descriptive level, there were more letter task choices for easy than hard letter stimuli when the preceding task was the letter task (i.e., task repetition), but this difference was numerically smaller when the preceding task was the color than the letter task.

#### Task performance – Effects of letter difficulty under free and forced letter-task processing

**Fig. 7:**
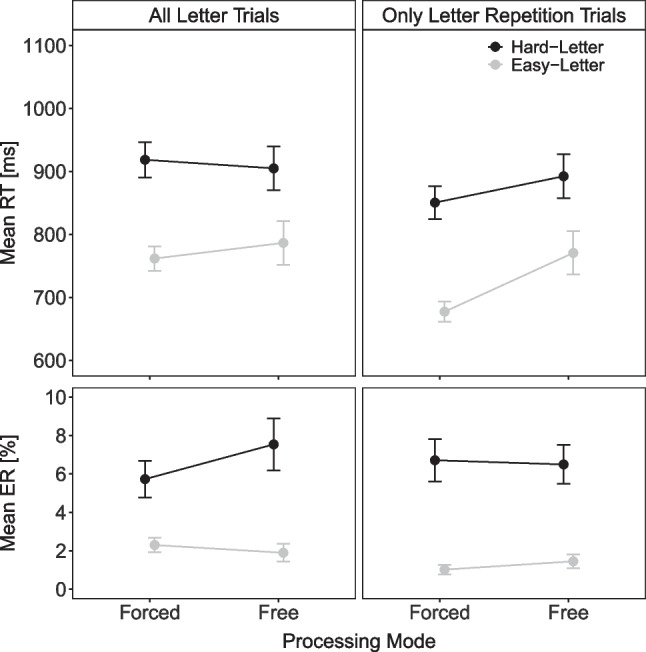
Task performance in Experiment 3. Note. Mean reaction time (RT) and mean error rate (ER) of letter task responses in Experiment 3 as a function of letter task difficulty (easy, hard) and processing mode (free, forced), including all letter task trials (*left column*) and only letter task trials preceded by letter task trials (*right column*)

Figure 7 shows the mean RT and mean PE for letter task responses as a function of letter task difficulty (easy, hard) and processing mode (free, forced). A 2$\times$2 within-subject ANOVA on mean RT revealed a significant main effect of letter difficulty, indicating faster responses with easy than hard letter stimuli (760 vs. 894 ms), *F*(1, 57) = 176.37, $p < .001, \eta ^2_p$ = .76 (with *p* = .818 and $\eta ^2_p<$ .01 for the main effect of processing mode). A significant interaction indicated that the letter difficulty effect was smaller under a free- than forced-choice processing mode (119 vs. 156 ms), *F*(1, 57) = 9.96, *p* = .003, $\eta ^2_p$ = .15. The ANOVA on mean PE also revealed a significant main effect of letter difficulty with less erroneous responses with easy than hard letter stimuli (2.1% vs. 6.6%), *F*(1, 57) = 20.51, $p < .001, \eta ^2_p$ = .26. The interaction was only marginally significant, but descriptively there was a larger letter difficulty effect under free-choice compared to forced-choice processing (5.6% vs. 3.4%), *F*(1, 57) = 4.88, *p* = .046, $\eta ^2_p$ = .15. Note than when combining RT and error rates using the IES, the ANOVA on the IES revealed no significant interaction, *F*(1, 56) = 0.04, *p* = .840, $\eta ^2_p<$ .01. The main effect of processing mode was not significant (*p* = .226, $\eta ^2_p$ = .03). Thus, there were again a reduced difficulty effect in mean RTs under a free-choice compared to a forced-choice mode. Interestingly, this effect was in the opposite direction for error rates and was not significant when combining RTs and error rates, suggesting that the benefit of choice on performance (in terms of reduced difficulty effect) is restricted to RTs.

We again reran the performance analyses, focusing exclusively on (letter) task repetition trials (see Fig. 7). For mean RT, the ANOVA revealed again a significant main effect of letter difficulty with faster responses with easy than hard letter stimuli (724 vs. 871 ms), *F*(1, 57) = 166.80, $p < .001, \eta ^2_p$ = .75. The main effect of processing mode was also significant, with slower responses under a free- than forced-choice processing mode (831 vs. 764 ms), *F*(1, 57) = 9.51, *p* = .003, $\eta ^2_p$ = .14. The interaction was significant reflecting again a reduced letter difficulty effect under a free- than forced-choice processing mode (121 vs. 173 ms). *F*(1, 57) = 12.09, *p* = .001, $\eta ^2_p$ = .17. For mean PE, there was only a significant main effect of letter task difficulty with fewer errors with easy than hard letter stimuli (1.6% vs. 6.6%), *F*(1, 57) = 32.94, $p<$ .001, $\eta ^2_p$ = .58 (all other $p > .411, \eta ^2_p<$ .02). In sum, as with the previous two experiments, the results of these additional analyses again revealed that the smaller difficulty RT effect under a free-choice compared to a forced-choice processing mode persists even when considering only repetition trials. Moreover, these analyses also demonstrate that this benefit of choice on task performance is present without any evidence of a speed–accuracy tradeoff, as seen in the overall analysis.

## General discussion

In this study, we examined how alterations in the processing difficulty of task-specific information affect task processing in settings where tasks are either freely chosen or assigned. Specifically, the perceptual processing difficulty was manipulated in Experiments 1 and 2 by altering the proportion of colored dots and the processing difficulty during decision-making was manipulated in Experiment 3 by varying the mental rotation of letter stimuli. Across all experiments, we found that both free-choice and forced-choice processing were more impaired when performing tasks in harder conditions than in easier ones. However, the most critical novel finding was that the difficulty effect on task performance was generally less pronounced under free-choice than under forced-choice processing. The reduced sensitivity to the difficulty of the presented information when choosing a task freely suggests that having personal control over task selection can lead to more stable information processing.

To account for the observed behavioral advantage in terms of a reduced difficulty effect in free-choice processing, it is necessary to postulate some differences between processing information in free-choice versus forced-choice scenarios. In general, research from various domains indicates that the internal representations of free- and forced-choice task goals differ to some extent (e.g., Qiao et al., 2023; Forstmann et al., 2006; Orr and Banich, 2014). For example, the results might be explained by motivational accounts such as self-determination theory, according to which higher motivation when people choose their own goals can have positive effects on their performance (e.g., Deci et al., 1989; Ryan and Deci, 2000; Bouzidi et al., 2022). Specifically, freely chosen task goals might be more robustly internally represented, and this enhanced task focus makes participants less susceptible to fluctuations in the difficulty of the information presented. Of course, other explanations within motivational (or non-motivational) theories are possible, but the general point is that the present study provides new empirical evidence that choosing a task can benefit task performance.

For example, considering also the task choices in free-choice trials, it seems especially useful to consider performance optimization accounts (e.g., Miller et al., 2009; Leonhard et al., 2011; Mittelstädt et al., 2019). Specifically, across all experiments, participants showed a general preference for the task with easier stimuli compared to harder ones.^4^ As the difficulty of stimuli for one of the two tasks varied randomly adjustments in task choices in response to processing difficulties have to take place during a trial (i.e., after the onset of the stimuli). Studies employing dual-task paradigms show that it is, in principle, possible to process two tasks in parallel (e.g., Mittelstädt et al., 2022; Pashler, 1994; Mackenzie and Leuthold, 2011; Mittelstädt et al., 2017). Thus, in the free-choice trials of the present study, it seems possible that processing for the two tasks proceeds in parallel and that participants determine which task has made more progress before completing the processing of the more advanced task. As a result, they tend to select a task associated with a more difficult stimulus only in trials where processing of this stimulus was more advanced, leading to a generally smaller difficulty effect in free-choice modes, as such performance optimization is not possible in a forced-choice mode. This performance optimization account also fits with the performance in the free-choice task involving consistent difficulty (i.e., the letter task in Experiments 1 and 2, and the color task in Experiment 3). Specifically, as revealed by exploratory analyses illustrated in the appendix, performance on the task without difficulty manipulation was worse with a hard color stimulus than with an easy one. This suggests that the stimulus of the non-chosen task was processed to some degree in parallel with the letter task, using more of the limited processing resources when it was more difficult (for evidence of perceptual and central interference in dual tasks, see e.g., Duncan et al., 2021; Brisson and Jolicoeur, 2007; Mittelstädt et al., 2017).

In future studies, it will be beneficial to delve deeper into the potential factors influencing the comparison between free-choice and forced-choice task processing. For example, it would be useful to investigate how the current results hold up when applying a voluntary task choice procedure that separates task choice from task processing (e.g., Mittelstädt et al., 2018; Arrington and Logan, 2005; Spitzer et al., 2022). Specifically, participants would first choose between two tasks during a choice phase and then be presented with a task-specific stimulus with varying difficulty. According to performance optimization accounts, this approach might lead to similar difficulty effect in free-choice compared to forced-choice trials. According to motivational accounts, participants might still be more motivated in free-choice trials so this enhanced motivation could lead to more stable information processing and, hence, a reduced difficulty effect in free-choice trials. However, if participants select a task without seeing the actual stimulus, they might feel they have less control over the outcome – a factor that could be crucial in valuing free choices (e.g., Mühlberger et al., 2017; Ly et al., 2019).

The performance and choice patterns were generally consistent across experiments, suggesting that the results can be generalized across difficulty changes localized to different processing stages (i.e., perception and decision). However, it is interesting to note that exploratory between-experiment analyses reported in Appendix B showed that the difficulty effect in task choice behavior was smaller in Experiment 3 (decision difficulty) compared to Experiments 1 and 2 (perceptual difficulty). Although the findings of these post hoc analyses need to be treated with caution and require direct testing, it seems reasonable to speculate about why the type of manipulation might matter. As mentioned above, integrating characteristics of stimulus processing into task choices requires determining the processing difficulty associated with a task prior its final processing. Because the difficulty arises later in processing in Experiments 3, participants might have often already chosen the task that requires processing of a hard stimulus, so that reconsidering and switching to the alternative task would mean wasting the progress made in the task so far. Furthermore, this might explain why participants in this experiment generally displayed a strong preference for the color task with consistent difficulty, with a significant number being excluded due to the absence of any letter task trials. Because it is more challenging for participants to adjust their choice behavior in response to the unpredictable difficulty of the letter task, some participants employed a proactive global task choice strategy, deciding to always choose the color task whenever possible – a strategy that helped them avoid the more difficult letter stimuli (for further discussion into individual differences in the use of global task choice strategies see Mittelstädt et al., 2021).^5^

In general, several researchers assume that the processes used for performing an individual task can be subdivided into three successive component stages (i.e., perception, response selection, and response execution, cf. Pashler, 1984; Rubinstein et al., 2001). Considering that it was possible for participants to adapt their choices to processing difficulties arising after perceptual processing is completed suggests that a task can be chosen even when participants are already engaged in some central processing. Future studies could further investigate how voluntary choices are affected by manipulations of difficulty related to response execution. For example, consider unpredictable changes in motor demands, such as trials requiring weak forces in some trials and strong forces in others to press keys for the task to which this manipulation is applied. Since a response is presumably only initiated after a response is selected, determining the motor difficulty takes place quite late. Thus, participants might have already fully committed to the task by this stage and hence do not adapt their choices to the motor manipulation. Additionally, researchers assume that task switching requires additional reconfiguratory processes for activating and implementing a new task (e.g., Koch et al., 2018; Rogers and Monsell, 1995). It would be interesting to better understand how these processes are affected by stage-specific processing manipulations. For example, it is often assumed that the first component of reconfiguration can be completed before stimulus presentation, whereas the second component of reconfiguration can only be triggered by a task-specific stimulus (e.g., Koch et al., 2018; Rubinstein et al., 2001). Given that stimulus difficulty varied unpredictably in the present study, it seems particularly critical to consider the second component, as preparatory processes before a trial are unlikely to differ between the easy and hard conditions.

Finally, it may also be useful to more directly investigate the extent to which comparisons between free and forced-choice behavior depend on the options available to participants. For example, in the present study, participants could choose among forced different cognitive tasks, while in other settings, participants choose between responses within a single task (e.g., red_forced_ = left key, green_forced_ = right key, blue_free_ = left or right key, cf. Naefgen et al., 2018; Berlyne, 1957; Naefgen and Janczyk, 2019). Although the act of choosing a task is arguably different from selecting a response, certain similarities might exist (e.g., Naefgen et al., 2022). For example, it appears that choices in both scenarios might be influenced by a desire to optimize performance (e.g., by selecting the easier task if possible) (e.g., Naefgen et al., 2022) Furthermore, free-choice responses are often slower than forced-choice responses (e.g., Naefgen et al., 2018; Naefgen and Janczyk, 2018; Berlyne, 1957), which fits with the present study where free-task choice responses were slower than forced-task choice responses when controlling for task transition (i.e., looking only at task repetitions), suggesting that in both cases, time-consuming choice processes take place. More generally, while the present study shows a benefit of task choice on performance in terms of reduced difficulty effects in free-choice compared to forced-choice tasks, this exploratory finding also reflects a general performance disadvantage of choosing freely between tasks. Thus, in future studies, it also seems useful to more directly test whether responding in free-choice tasks can be more strongly slowed down relative to forced-choice tasks when choosing becomes more time-consuming (e.g., by choosing between four tasks instead of two). In any case, we hope that the approach in the present study, along with its empirical findings, further highlights the usefulness of jointly studying processing under free versus forced task goals to shed more light on the factors and mechanisms that are special to free choice behavior.

### Open practice statement and availability of data and materials

Preregistrations for Experiments 2 and 3 are available via the Open Science Framework at https://osf.io/twqxd and https://osf.io/e47v2, respectively.

Raw data of all experiments are available via the Open Science Framework at https://osf.io/w7t43/. Materials for the experiments reported here are available from the authors upon request.

## Data Availability

The code used to analyze the data is available upon request.

## Appendix A Additional free-choice task performance analyses

To gain deeper insight into how participants might adapt their task choice behavior in response to stimuli of varying difficulty, we also explored task performance in trials where participants selected the task with consistent difficulty. Specifically, since in each experiment we varied the difficulty of the stimuli for only one of the two tasks, we could investigate in free-choice trials how the presence of the easy versus hard stimuli affects processing of stimuli associated with the task with consistent difficulty (i.e., Experiments 1 and 2: letter task; Experiment 3: color task). Intuitively, this task should not be influenced by the difficulty manipulation associated with another task. However, as noted earlier, the two stimuli might be at least partially processed in parallel. Therefore, the concurrent processing of a hard stimulus – as compared to an easy one – of the non-chosen task, alongside the chosen task, might consume more of the limited processing resources, resulting in a larger slowdown of processing the chosen task with constant difficulty in the presence of a hard stimulus compared to an easy one.

**Table 1:** Performance of the task without difficulty manipulation

	Experiment 1: Letter task		Experiment 2: Letter task		Experiment 3: Color task	
Measure	Easy color	Hard color	Easy color	Hard color	Easy letter	Hard letter
**Mean RT**	815 (25)	881 (28)	703 (18)	745 (24)	712 (40)	735 (41)
**Mean PE**	3.3 (0.8)	3.2 (0.5)	2.8 (0.5)	2.6 (0.5)	2.9 (0.3)	2.2 (0.7)

**Table 2:** Performance of the task without difficulty manipulation, considering only task repetitions

	Experiment 1: Letter task		Experiment 2: Letter task		Experiment 3: Color task	
Measure	Easy color	Hard color	Easy color	Hard color	Easy letter	Hard letter
**Mean RT**	777 (24)	823 (29)	678 (18)	702 (23)	672 (38)	687 (41)
**Mean PE**	3.3 (0.8)	3.4 (0.9)	2.7 (0.4)	2.3 (0.5)	2.3 (0.3)	1.6 (0.2)

### Experiment 1: Effects of color difficulty on free letter-task performance

Given the unpredictably changing difficulty of color task stimuli across trials, the preference for the color task with easy color stimuli suggests that participants engaged in processing during a trial before they then reactively adjusted their choice behavior based on which task was progressing more. Thus, some concurrent preliminary parallel (resource-limited) task processing may take place before one of the tasks is actually selected. If this is the case, performance of the letter task in free choice trials might be affected by the presence of the specific color stimulus. Indeed, letter task responses were significantly faster when the color task stimulus was easy than when it was hard (815 vs. 881 ms), *t*(42) = 6.31, $p<$ .001, *d* = 0.97 (see also Table 1). There was no evidence that mean PEs of letter task responses differed between easy versus hard color stimuli (3.3% vs. 3.2%, *p* = .919). Thus, this effect suggests that participants at least partially process both stimuli in parallel, with a hard color stimulus, compared to an easy one, withdrawing more of the limited cognitive resources away from processing the letter task.

We also checked whether this effect held true when only considering letter task repetition trials for two reasons: First, in these trials, the potential additional impact of switch-related cognitive processes is eliminated. Second, participants in letter switch trials may generally be inclined to process the color task first before switching to the letter task. Thus, if the effect held true when focusing only on repetition letter trials, it would provide even stronger evidence that the effect arises due to parallel processing. The pattern was similar when only considering letter task repetition trials (see also Table 2). Specifically, we again observed significantly faster letter task responses when the color task stimulus was easy than when it was hard (777 vs. 823 ms), *t*(42) = 2.69, *p* = .010, *d* = 0.42, and no significant difference in error rates between easy and hard stimuli (2.7% vs. 2.3%, *p* = .364).

### Experiment 2: Effects of color difficulty on free letter-task performance

Letter task performance in free choice trials was again affected by the presence of the specific color stimulus. Specifically, letter task responses were significantly faster when the color task stimulus was easy than when it was hard (704 vs. 745 ms), *t*(41) = 5.17, $p<$ .001, *d* = 0.79. There was no evidence that mean PEs of letter task responses differed between easy versus hard color stimuli (2.8% vs. 2.6%, *p* = .651). This pattern held true when only considering letter task repetition trials: We again observed significantly faster letter task responses when the color task stimulus was easy than when it was hard (678 vs. 702 ms), *t*(41) = 3.03, $p<$ .004, *d* = 0.47, and no significant difference in error rates between easy and hard stimuli (2.7% vs. 2.3%, *p* = .364).

### Experiment 3: Effects of letter difficulty on free color-task performance

We again investigated whether the performance when selecting the task with equal difficulty (i.e., the color task) is influenced by the difficulty of the stimulus associated with the non-chosen task (i.e., the letter task stimulus). Color task responses were significantly faster when the letter task stimulus was easy than when it was hard (712 vs. 735 ms), *t*(57) = 3.55, $p<$ .001, *d* = 0.47. Mean PEs of color task responses were slightly larger when the color stimulus was easy than when it was hard (2.9% vs. 2.2%), but this difference was only marginally significant, *t*(57) = 1.77, *p* = .081, *d* = 0.23. When only considering color task repetition, color task responses were also significantly faster for easy versus hard letter stimuli in either RTs (672 vs. 687 ms), *t*(57) = 2.20, *p* = .032, *d* = 0.29. While error rates were slightly larger for easy than hard stimuli (2.3% vs. 1.6%), this difference was only marginally significant, *t*(57) = 1.89, *p* = .064, *d* = 0.25.

## Appendix B Additional between-experiment comparisons

To check for between-experiment differences, we conducted additional exploratory (not preregistered) analyses.

Specifically, we conducted a 2$\times$3 mixed ANOVA with difficulty (easy, hard) as a within-subject factor and experiment (Experiments 1, 2, 3) as a between-subject factor, analyzing the percentage of trials in which participants chose the task for which the difficulty manipulation was applied (i.e., the color task in Experiments 1 and 2, and the letter task in Experiment 3). The ANOVA revealed a significant main effect of difficulty, reflecting a preference for selecting the task with easier rather than harder stimuli (39.80% vs. 33.54%), *F*(2, 140) = 56.35, $p<$ .001, $\eta ^2_p$ = .29. The interaction between difficulty and experiment was also significant, *F*(2, 140) = 11.84, $p<$ .001, $\eta ^2_p$ = .14 (with *p* = .180 and $\eta ^2_p$ = .02 for the main effect of experiment). Follow-up 2$\times$2 mixed ANOVAs conducted for each pair of experiments indicated that this interaction was primarily driven by a reduced difficulty effect in Experiment 3. Specifically, the ANOVA comparing Experiments 1 and 2 showed that the interaction was only marginally significant, *F*(1, 83) = 3.0, *p* = .087, $\eta ^2_p$ = .03. However, the interaction was significant when comparing Experiments 1 and 3, *F*(1, 99) = 19.79, $p<$ .001, $\eta ^2_p$ = .17, and also when comparing Experiments 2 and 3, *F*(1, 98) = 15.58, $p<$ .001, $\eta ^2_p$ = .14.

We also checked for differences in the difficulty effect on task performance between experiments. Specifically, we also conducted a 2$\times$2$\times$3 mixed ANOVA with difficulty (easy, hard), processing mode (free, forced) as within-subject factors and experiment (Experiments 1, 2, 3) as a between-subject factor on mean RT and mean ER. For mean RT, there were significant main effects of difficulty, *F*(1, 140) = 451.51, $p<$ .001, $\eta ^2_p$ = .76, and processing mode, *F*(1, 140) = 7.60, $p<$ .00, $\eta ^2_p$ = .76 (with *p* = .057 and $\eta ^2_p$ = .04 for the main effect of experiment). The interaction between processing mode and experiment, *F*(1, 140) = 3.59, *p* = .030, $\eta ^2_p$ = .05, as well as the interaction between processing mode and difficulty were significant, *F*(1, 140) = 43.64, $p<$ .001, $\eta ^2_p$ = .24. Interestingly, the interaction between difficulty and experiment was not significant (*p* = .470 and $\eta ^2_p$ = .01), suggesting that there was no evidence that the difficulty effect was larger in one experiment (with *p* = .059 and $\eta ^2_p$ = .04 for the three-way interaction). For mean PE, there was a significant main effect of difficulty, *F*(1, 140) = 47.76, $p<$ .001, $\eta ^2_p$ = .25 (with *p* = .084 and $\eta ^2_p$ = .03 for the main effect of experiment and *p* = .365 and $\eta ^2_p$ = .01 for the main effect of processing mode). The interaction between processing mode and experiment, *F*(1, 140) = 72, *p* = .027, $\eta ^2_p$ = .05, as well as the interaction between processing mode and difficulty were significant, *F*(1, 140) = 5.06, *p* = .026, $\eta ^2_p$ = .05. However, the interaction between difficulty and experiment was only marginally significant, *F*(2, 140) = 2.93, *p* = .057, $\eta ^2_p$ = .04 (with *p* = .315, $\eta ^2_p$ = .02 for the three-way interaction).

In sum, the results from these exploratory analyses suggest that the difficulty effect in biasing choices was smaller in Experiment 3 than in Experiments 1 and 2. However, there was limited, if any, evidence that the difficulty effect on task performance was larger in Experiment 3 compared to the other experiments. Thus, this might suggest that the type of manipulation (i.e., mental rotation versus perceptual degradation), rather than the strength of the manipulation on task performance, is critical in affecting task choice behavior. However, as these post hoc between-experiment analyses of independently planned studies were exploratory and other differences might play a role (e.g., time point of the experiment), these exploratory findings need to be treated with caution.

## Footnotes

1 Note that in all experiments the results were similar when applying a stricter RT outlier cutoff (e.g., 2500, 2000, 1500 ms) and when including post-error trials.

2 We then also separately examined the number of trials for color RT analyses for each participant in each condition (free/forced X easy/hard) to ensure that each participant had an adequate number of trials for a reliable estimate of condition-specific RTs. Two participants in Experiment 2 had four and nine trials in one condition, while all other participants had over ten trials in each condition. Excluding the two participants with less than ten trials did not impact the results.

3 Although the interaction on mean PE was relatively small, its pattern was opposite to that observed for mean RT. To address this speed–accuracy tradeoff, we combined RT and error rates using the inverse efficiency score (IES, cf. Townsend & Ashby, 1983). The ANOVA on the IES revealed a significant interaction, indicating a smaller difficulty effect under free-choice processing compared to forced-choice processing. *F*(1, 42) = 13.23, $p < .001, \eta ^2_p$ = .24.

4 Note that the performance pattern seems incompatible with a pure practice-related explanation. Specifically, in free-choice trials, participants selected the respective task with easy stimuli more frequently than with hard stimuli, whereas in forced-choice trials, they performed the task equally often with both easy and hard stimuli. Thus, participants practiced processing the difficult stimulus less frequently than the easy one in a free-choice mode and these mode-specific practice differences alone should actually lead to a larger difficulty effect under free-choice than under forced-choice processing.

5 Considering that the letter task in the easy condition of Experiment 3 was even easier to process (i.e., with a smaller rotation angle) than in Experiment 1 and 2, where only a few participants had to be excluded for strongly avoiding the letter task, it seems unlikely that this task was too difficult for participants in the easy condition of Experiment 3.
